# Visualizing fungal metabolites during mycoparasitic interaction by MALDI mass spectrometry imaging

**DOI:** 10.1002/pmic.201500510

**Published:** 2016-04-13

**Authors:** Matthias Holzlechner, Sonja Reitschmidt, Sabine Gruber, Susanne Zeilinger, Martina Marchetti‐Deschmann

**Affiliations:** ^1^ Institute of Chemical Technologies and AnalyticsVienna University of TechnologyViennaAustria; ^2^ Institute of MicrobiologyUniversity of InnsbruckInnsbruckAustria; ^3^ Institute of Chemical EngineeringVienna University of TechnologyViennaAustria

**Keywords:** Fungal communication, MALDI MSI, Mycoparasitism, Peptaibiotics, Rhizoctonia, Trichoderma

## Abstract

Studying microbial interactions by MALDI mass spectrometry imaging (MSI) directly from growing media is a difficult task if high sensitivity is demanded. We present a quick and robust sample preparation strategy for growing fungi (*Trichoderma atroviride*, *Rhizoctonia solani*) on glass slides to establish a miniaturized confrontation assay. By this we were able to visualize metabolite distributions by MALDI MSI after matrix deposition with a home‐built sublimation device and thorough recrystallization. We present for the first time MALDI MSI data for secondary metabolite release during active mycoparasitism.

## Technical brief

We present that mass spectrometry imaging (MSI) is capable of visualizing the release of secondary metabolites upon host sensing and fungal interaction directly from a miniaturized potato‐dextrose agar (PDA) platform set up for a confrontation assay.

Fungi are globally distributed and numerously occurring microorganisms exceeding an estimated total number of 1.0 to 1.5 million 1. Besides their use as producers and agents for pharmaceuticals, enzymes, organic acids and foods 2 the fungal kingdom plays an essential role in natural ecosystems and also comprises pathogenic and parasitic species. Fungal phytopathogens like *Rhizoctonia spp*. account for 80% of plant diseases 3 and demand for sustainable, ecofriendly strategies to prevent crop loss. Several fungal species are specialized to attack and antagonize plant pathogens by impeding their growth or directly killing the pathogens. Such beneficial microorganisms are already commercially applied as biological control agents (BCAs) 4. The fungus *T. atroviride* can establish itself in the plant rhizosphere, thereby increasing plant growth and productivity 5, 6. In addition to that, *Trichoderma* is able to directly parasitize a range of pathogens (e.g. *Rhizoctonia solani*) by a process called mycoparasitism, allowing for the effective use of *Trichoderma* as BCA 7. The processes leading to mycoparasitism are rather complex, yet sensing of the host and growth towards it, the active release of small molecules and enzymes and the subsequent penetration and killing of the host are key events. It is already known that *Trichoderma* produces secondary metabolites contributing to the antagonistic potential towards plant pathogens 8, 9, 10, and peptaibiotics are one important group of significant concentration 11, 12. Within peptaibiotics, peptaibols represent a subgroup of non‐ribosomal, membrane‐active linear chained polypeptides characterized by a C‐terminal alcohol residue, an acylated N‐terminus and a high level of unusual amino acids, like α‐aminoisobutyric acid (Aib), 2‐isovaline (Iva), and hydroxyproline (Hyp) 13, 14. Up to now peptaibols were detected either from fungal spores by intact cell mass spectrometry (ICMS) 15 or by well‐established HPLC‐MS/MS methods after isolation from cell cultures 14, 16, 17. Based on work using MSI in the field of microbiology 18, our objective was to develop a sample preparation method suitable for studying *Trichoderma‐Rhizoctonia* interactions directly from cultivation to visualize metabolite distributions induced by mycoparasite‐host interaction to build a solid basis for future work in the field of fungal communication.

For our experiments we used chemicals of highest grade (supporting information). For the mycoparasitic *T. atroviride*‐*R. solani* interaction, a miniaturized confrontation assay had to be established on conductive indium tin oxide (ITO) slides. *T. atroviride* and *R. solani* were first grown separately in petri dishes on PDA at 28°C. Then, an ITO slide was covered with a thin layer of PDA (approx. 400 μm) and mycelia plugs of each fungus were deposited on the glass slide approx. 1–2 cm apart and incubated at 28°C until (i) hyphae nearly reached each other (approx. 2–4 mm apart) or (ii) were clearly interacting which was monitored with a light microscope. After approx. 1–2 days, when fungal hyphae were grown towards each other, the sample was removed from the incubator and immediately stored at –70°C. For MALDI MSI the glass slides were removed from the freezer and thawed in a vacuum desiccator for 2 h. Matrix for MALDI MSI was deposited with a home‐built sublimation apparatus in a vacuum‐sealed, pressure‐stabilized deposition chamber with constant heating for matrix vaporization and controlled sample plate cooling for deposition. 2,5‐dihydroxy benzoic acid (2,5‐DHB) dissolved in acetone was sublimed at 120°C and 35 mTorr to gain a matrix layer of 0.3 mg/cm². Recrystallization and hydration was done according to Yang et al. 19. Finally, slides were dried for 2 min at 85°C and immediately used for analysis. Imaging measurements were performed on a MALDI TOF/RTOF mass spectrometer (ultraflextreme™, Bruker Daltonics, Bremen, Germany) in a mass range from 100 to 2600 Da with ion suppression for ions below 50 Da. Images were normalized to the total ion current (TIC) (for full details on matrix deposition and MS analysis see Supporting Information).

MALDI MSI for PDA grown fungi is not straightforward. First metabolism has to be stopped, which was facilitated by immediate storing the sample at –70°C. Furthermore, the water content of the PDA layer had to be removed to prevent the vacuum system of the mass spectrometer from crashing. For this, samples were removed from the freezer and thawed while drying in a vacuum desiccator for 2 h. Then the glass slides were introduced into the fine vacuum of the sublimation unit guaranteeing completely dry samples for analysis.

**Figure 1:**
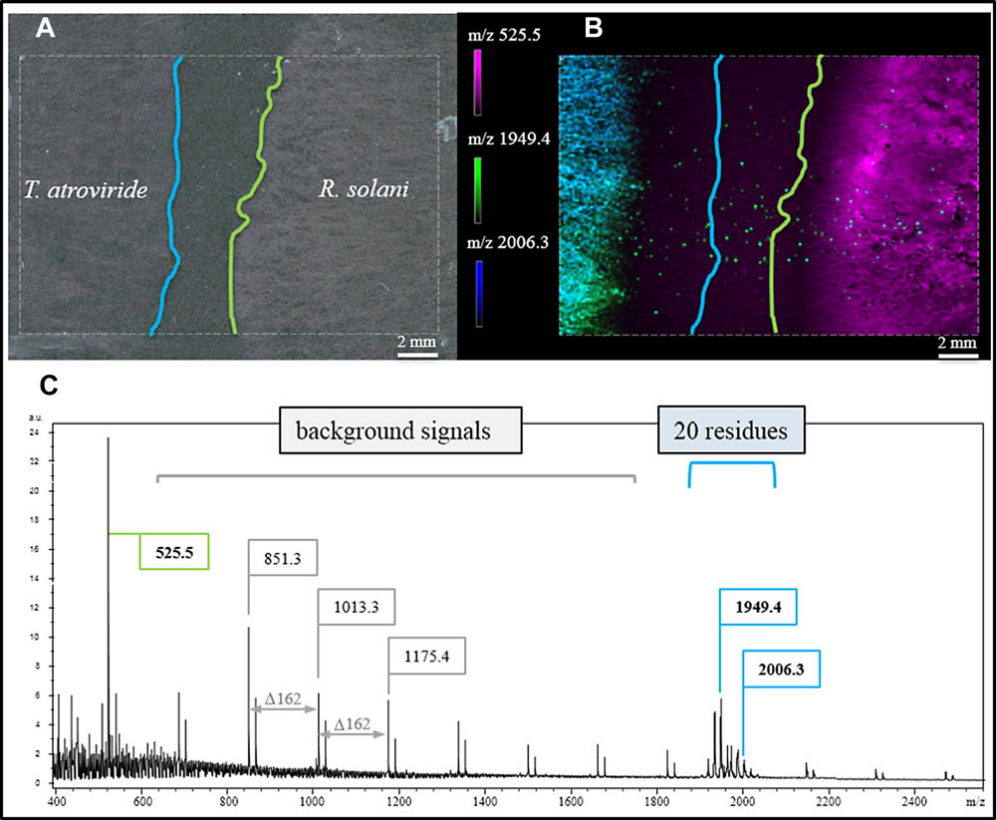
MALDI MSI of physically non‐interacting *T. atroviride* and *R. solani* hyphae. (A) Light microscopic image showing non‐interacting hyphae. (B) Distribution of selected *m/z* values representing characteristics for *T. atroviride* (*m/z* 1949.4 and 2006.3) and *R. solani* (*m/z* 525.5) (Supporting Information S1). (C) Mass profile for the selected ROI (outlined rectangle) exhibiting potato dextrose agar derived background signals with Δ*m/z* 162 and signals characteristic for each fungus (green … *R. solani*, blue … *T. atroviride*). Blue and green lines in (A) and (B) mark the outer rim of hyphal growth.

**Figure 2:**
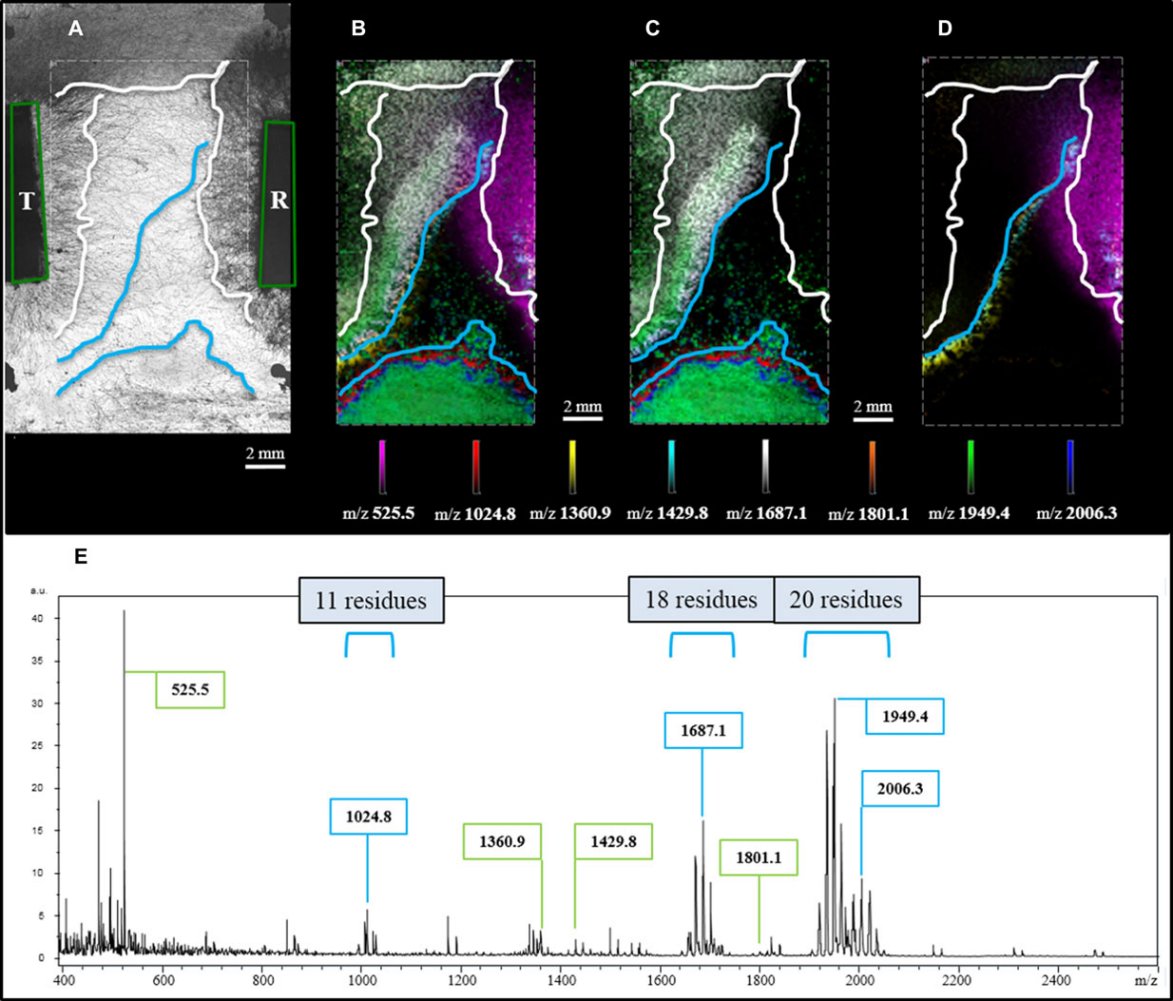
MALDI MSI of physically interacting *T. atroviride* (T) and *R. solani* (R) hyphae. (A) Light microscopic image showing points of inoculation (green tetragons), the outer rim of hyphal growth for both species (white lines) and borders for features detected by MSI (blue lines). (B) Molecular distributions of selected secondary metabolites localized by MALDI MSI. (C) *T. atroviride* specific metabolites (Supporting Information S3). (D) *R. solani* specific metabolites (Supporting Information S4). (E) Profile mass spectrum exhibiting signals assigned to *R. solani* (marked in green) and *T. atroviride* (marked in blue).

Additionally, the hyphae‐covered PDA layer does not provide an even surface. Most critical is the point of inoculation where height differences can be up to 1 mm. Because the points of inoculation were of lesser interest, only areas of similar height were investigated (rectangular shaped regions of interest (ROIs) in Figs. 1 and 2) to reduce signal broadening to a minimum due to differences in flight times.

By this we were for the first time able to visualize analyte distributions characteristic for both fungi directly from agar‐covered ITO slides with good signal‐to‐noise (S/N) ratios (Fig. 1).

Figure 1A clearly shows that the fungal hyphae were not interacting (2–4 mm distance), yet already at this state characteristic analyte distributions could be determined (Fig. 1B). The exemplarily depicted distributions of *m/z* 525.5, 1949.4 and 2006.3 show that ions can specifically be assigned to either fungus. Interestingly, *m/z* 1949.4 and 2006.3 are present at apparent higher concentrations (higher signal intensities) in regions closer to the point of *T. atroviride* inoculation and almost not detected in the outer rim of the colony, while *R. solani* specific analytes were visible for the whole radially grown fungus. Yet *m/z* 1949.4 and 2006.3 seem to be released at an early growth stage to allow for migration towards *R. solani*. At this point we hypothesize that we have for the first time visualized metabolites released by *Trichoderma* during the phase of host sensing (details on single ion images see Supporting Information S1).

Compared with previous publications presenting antibiotic 20 or other chemical output 18 of fungi/bacteria grown on agar, this study presents signals with a very good S/N ratio (Fig. 1C), although background signals from PDA were not completely diminished (Supporting Information S2). We attribute this enhancement of detection to the fact that we established a miniaturized confrontation assay directly on glass slides, exhibiting therefore only a very thin layer of PDA providing nutrients. Even more important, matrix sublimation added the benefit of thorough sample drying and the rehydration step incorporated the analytes of interest very efficiently. At the current state of research we suspect the signals between *m/z* 1900 and 2000 to be representatives of above mentioned peptaibols because cultivation on solid media favors their production 21 and the observed *m/z* values correlate with data available in the peptaibiotics database (https://peptaibiotics‐database.boku.ac.at/ 22).

To strengthen our findings, MALDI MSI data were additionally collected from samples showing interacting hyphae (Fig. 2A). As can be seen in Fig. 2E metabolites were released to a high extent, suppressing the background signals from PDA. Figure 2B shows that the observed distributions correlate nicely with hyphal density. Again *m/z* 1949.4 and 2006.3 were observed, analytes considered to represent peptaibols containing 20 amino acid residues (details on single ion images see Supplemental S3). These m/z values are of high abundance also in an area far away from inoculation. This is caused by contamination coming from the inoculation procedure but not MALDI MSI sample preparation (Fig. 2B). This assumption is corroborated by light microscopy (Fig. 2A).

*m/z* 1024.8 and 1687.1 can be considered as peptaibols with chain lengths of 11 and 18 residues (Fig. 2C), from which 1024.8 was partially co‐localized with 2006.3, while 1687.1 showed high prevalence in areas of *T. atroviride* hyphae of lesser density. Taking a closer look at the distribution of *m/z* 1024.8 one may hypothesize that this particular metabolite is released simultaneously with or even earlier than *m/z* 2006.3. Especially its distribution in the area of contamination is corroborating this assumption. For *m/z* 1024.8 first MS/MS spectra exhibited mass differences supporting the presence of Aib, therefore confirming the hypothesis that some compounds are indeed peptaibols (data not shown). Metabolites with *m/z* values of 1360.9, 1429.8 and 1801.1 (Fig. 2D) are predominantly present at the borders of fungal interaction and are most likely released by *R. solani*, especially when considering their presence near the point of inoculation (details on single ion images see Supporting Information S4).

With this technical brief we show for the first time that MALDI MSI can be used to visualize metabolites released during *T.atroviride‐R. solani* interaction directly from PDA. First experiments show several distinct local distributions of metabolites for both, non‐interacting and interacting hyphae. Characteristic masses correlating to known peptaibols with chain lengths of 11, 18 and 20 residues were detected. We attribute this achievement to an enhanced sample preparation strategy facilitating a miniaturized confrontation assay carried out directly on glass slides. Furthermore, MALDI matrix application by sublimation in combination with thorough recrystallization/hydration allows good analyte extraction from the agar medium and incorporation into the matrix crystals.

Based on these basic technical improvements, future work will focus on (i) biological aspects to obtain a better understanding of fungal sensing mechanisms and communication, and (ii) technical aspects to increase mass spectral quality by decreasing height differences of growing hyphae and preserving conductivity for the agar‐coated targets.

*The authors have declared no conflict of interest*.

## Supporting information

As a service to our authors and readers, this journal provides supporting information supplied by the authors. Such materials are peer reviewed and may be re‐organized for online delivery, but are not copy‐edited or typeset. Technical support issues arising from supporting information (other than missing files) should be addressed to the authors.

Supporting Information on Chemicals and MALDI MSI Sample Preparation
Supplemental Figure S1
Supplemental Figure 2
Supplemental Figure 3
Supplemental Figure 4
Supplemental Figure S5 and S6
Supplemental Figure 7
