# Supplementary material for: Visualizing fungal metabolites during mycoparasitic interaction by MALDI mass spectrometry imaging
Source: Proteomics. 2016 Apr 13;16(11-12):1742–6. doi: 10.1002/pmic.201500510 (PMC4982067; doi:10.1002/pmic.201500510)
Supplement: Supplementary file 1 — Supporting Information on Chemicals and MALDI MSI Sample Preparation Supplemental Figure S1 Supplemental Figure 2 Supplemental Figure 3 Supplemental Figure 4 Supplemental Figure S5 and S6 Supplemental Figure 7 [file PMIC-16-1742-s001.docx]

**Supporting Information for Technical Brief**

VISUALIZING FUNGAL METABOLITES DURING MYCOPARASITIC INTERACTION BY MALDI MASS SPECTROMETRY IMAGING

M. Holzlechner^1^, S. Reitschmidt^1^, S. Gruber^2^, S. Zeilinger^2,3^, M. Marchetti-Deschmann^*1^

*^1^ Institute of Chemical Technologies and Analytics, Vienna University of Technology, Vienna, Austria;*

*^2^ Institute of Microbiology, University of Innsbruck, Innsbruck, Austria;*

*^3^ Institute of Chemical Engineering, Vienna University of Technology, Vienna, Austria*

**Supporting Information on Chemicals and MALDI MSI Sample Preparation:**

*Chemicals*

Glacial acetic acid and 2,5-dihydroxy benzoic (2,5-DHB) acid were obtained from Sigma Aldrich (St. Louis, MO, USA). Conductive indium tin oxide (ITO) glass targets were purchased from Delta Technologies (Loveland, CO, USA). MALDI MS calibrants, Angiotensin II, Neurotensin and the fragments Bradykinin 1-5 and ACTH 18-39, were obtained from LaserBio Labs (Sophia-Antipolis Cedox, F).

*MALDI matrix deposition*

Matrix was deposited with a home-built sublimation apparatus in a vacuum sealed, pressure controlled deposition chamber with constant heating for matrix vaporization and controlled sample plate cooling for deposition. 40.6 mg of 2,5-DHB per 7 mL acetone were placed in a preheated well (63 °C) forming a homogenous micro crystalline matrix layer after solvent evaporation. ITO slides with the dried fungi were mounted on the cooling plate and matrix was completely sublimed (120 °C, 35 mTorr). Amount of matrix coating per unit area was assessed with a balance and gave 0.3 mg/cm². Subsequent recrystallization and hydration according to Yang et al. [1] allowed analyte incorporation. Briefly, a filter paper soaked in 5 % aqueous acetic acid was placed in a glass petri dish. After attaching the matrix covered slide to a stainless steel plate and mounting it on the inner side of the cover, the petri dish was reassembled to form a hydration chamber and incubated in a constant temperature oven at 85 °C for 3 min. The slide was removed, dried at 85 °C for two minutes and immediately used for analysis.

*Imaging MALDI Mass Spectrometry*

Imaging measurements were performed on a MALDI TOF/RTOF mass spectrometer (UltrafleXtreme™, Bruker Daltonics, Bremen, Germany) using flexControl software v3.4. The Nd:YAG/355 nm smartbeam-II™ laser attenuator offset was adjusted to 65 % and laser fluency was operated at 55 %. Mass range was set from 100 – 2600 Da and ion suppression for ions below 50 Da. Data was collected at 0.50 Gs sampling rate. Pulsed ion extraction was adjusted to 120 ns. For reflectron positive ion mode, the source was set to an accelerating voltage of 20.00 kV with an extraction voltage of 17.95 kV, and a lens voltage of 7.50 kV. Imaging data were acquired with a spatial resolution of 120 µm by summing up 100 shots per array position without intraspot rastering using a laser repetition rate of 1 kHz. Mass spectra were calibrated externally using the cubic enhanced algorithm on the singly charged ions of 2,5-DHB (m/z 155.1), Angiotensin II (m/z 1046.5), Neurotensin (m/z 1673.9), Bradykinin fragment 1-5 (m/z 573.3) and ACTH 18-39 (m/z 2465.2).

*Image and Data Processing*

Image acquisition and imaging data processing were carried out using flexImaging software v3.0 (Bruker Daltonics). Top Hat algorithm was used to subtract the baseline of the average spectra. Images were normalized to the total ion current. Intensity bars in figures represent ion intensity scalings from 0 to 100%. In supplemental figures, single color and rainbow color codes were displayed for highlighting the spatial and intensity distributions of ions, respectively.

[1] Yang, J., Caprioli, R. M., Matrix Sublimation/Recrystallization for Imaging Proteins by Mass Spectrometry at High Spatial Resolution. *Anal. Chem. (Washington, DC, U. S.)* 2011, *83*, 5728-5734.

**SUPPLEMENTARY FIGURES**

**Supplemental Figure 1**


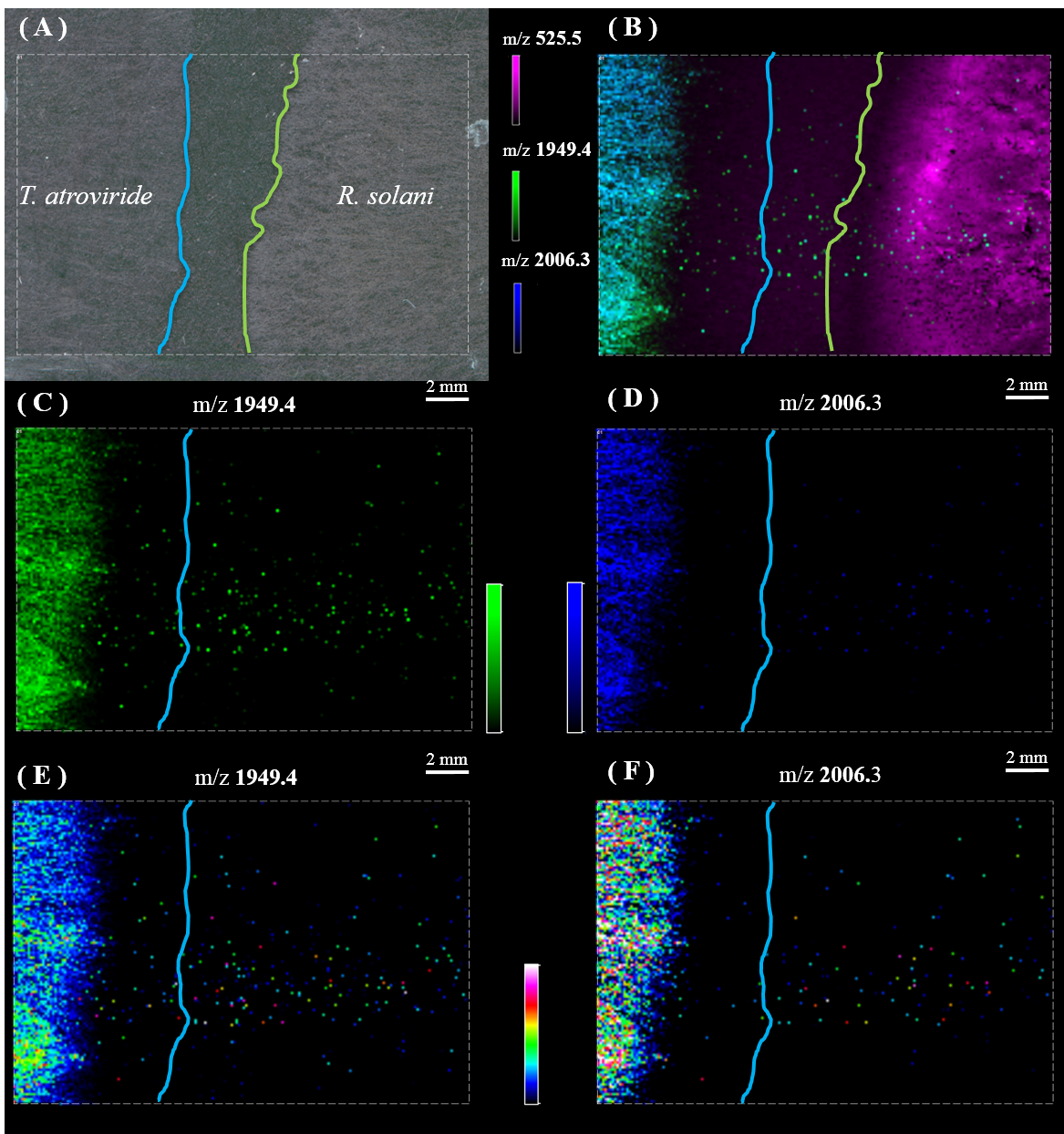


S1: MALDI MSI of physically non-interacting *T.atroviride* and *R.solani* hyphae. (A) Light microscopic image showing non-interacting hyphae. Blue and green lines mark the outer rim of hyphae growth. (B) Distribution of selected m/z values representing characteristics for *T.atroviride* (m/z 1949.4 and 2006.3) and *R.solani* (m/z 525.5). (C) and (D) visualize spatial distributed ions of m/z 1949.4 and m/z 2006.3. (E) and (F) show the respective ion intensity distributions.

**Supplemental Figure 2**


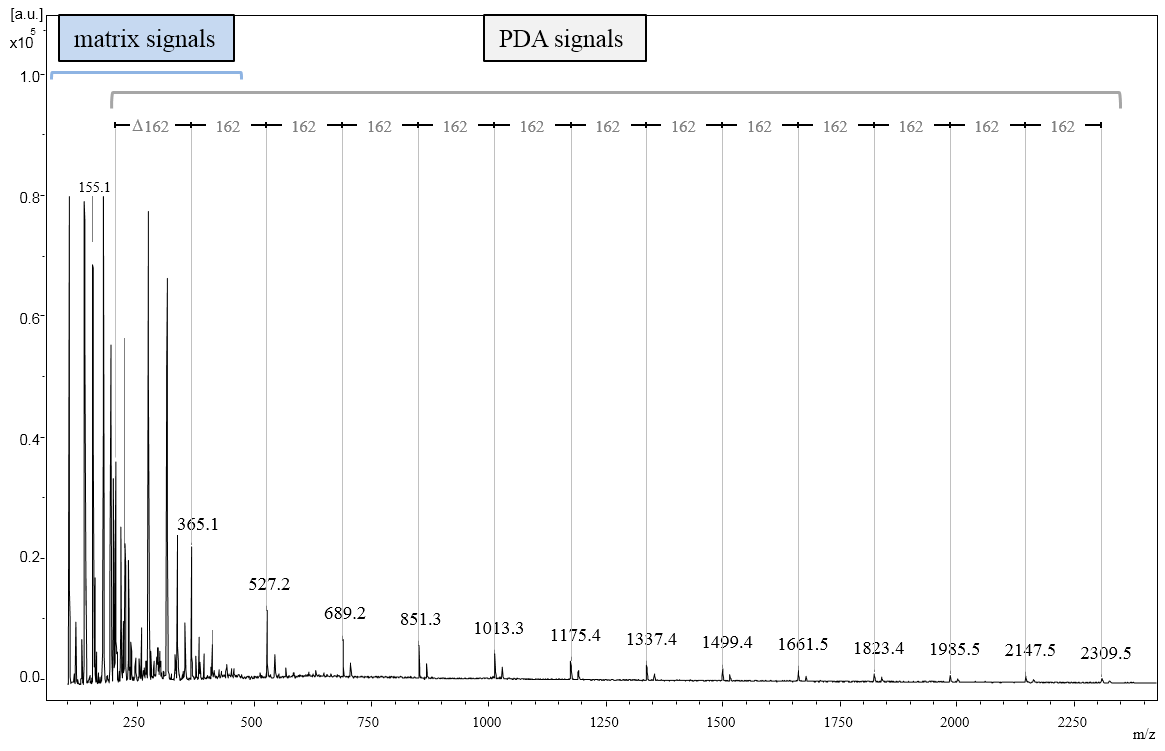


S2: Mass profile spectrum of potato dextrose agar exhibiting periodically occurring signals with Δm/z 162 in grey and 2,5-DHB derived matrix signals in blue (mass range 100-2300 Da).

**Supplemental Figure 3**


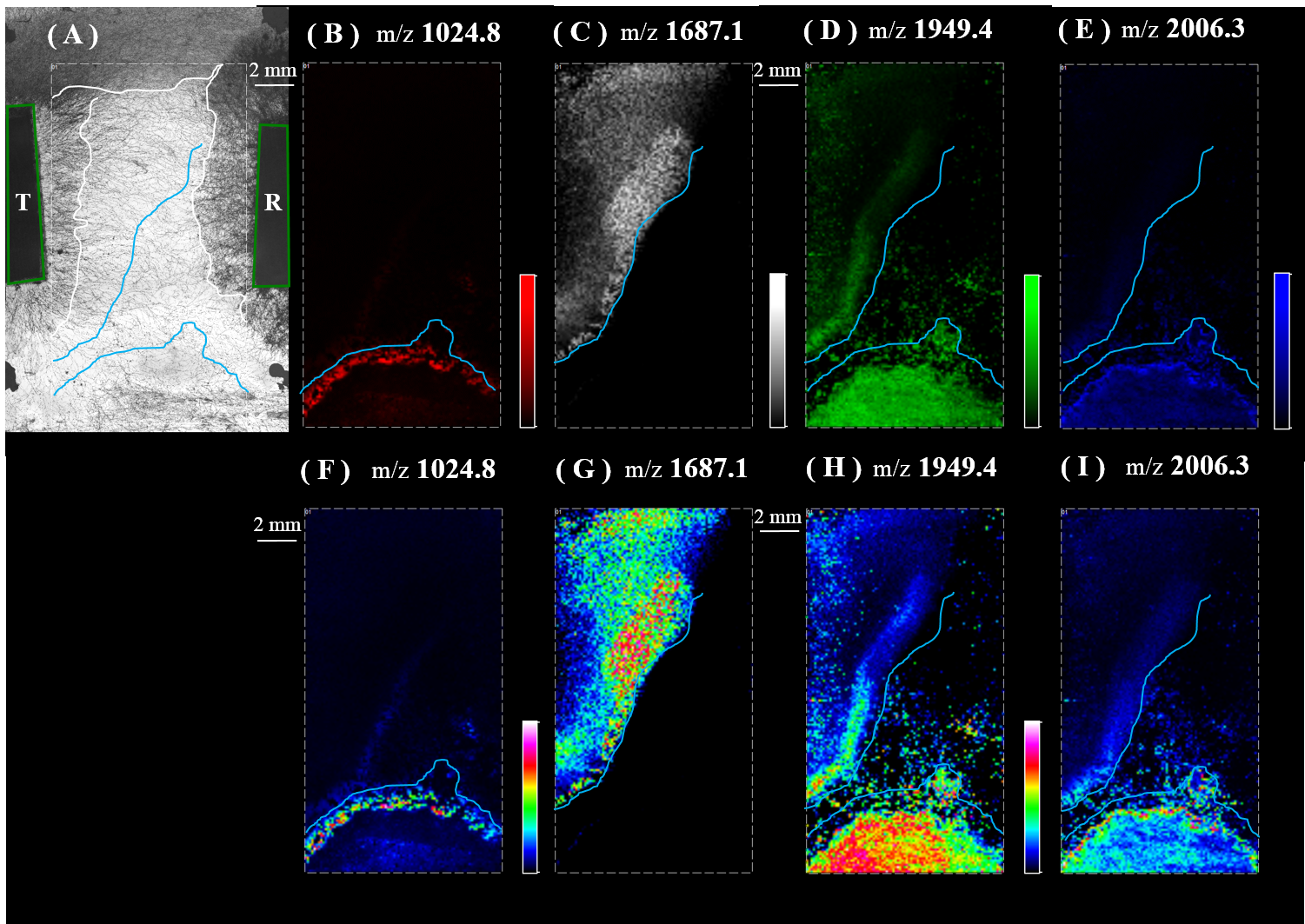


S3: Single ion images of *T.atroviride* (T) specific metabolites derived during physical interaction with *R.solani* (R). (A) Light microscopic image showing points of inoculation (green tetragons), the outer rim of hyphal growth for both species (white lines) and borders for features detected by MSI (blue lines). Spatial distributions of metabolites with (B) m/z 1024.8, (C) m/z 1687.1, (D) m/z 1949.4 and (E) m/z 2006.3 are visualized. Images (F), (G), (H) and (I) highlight the respective ion intensity distributions.

**Supplemental Figure 4**


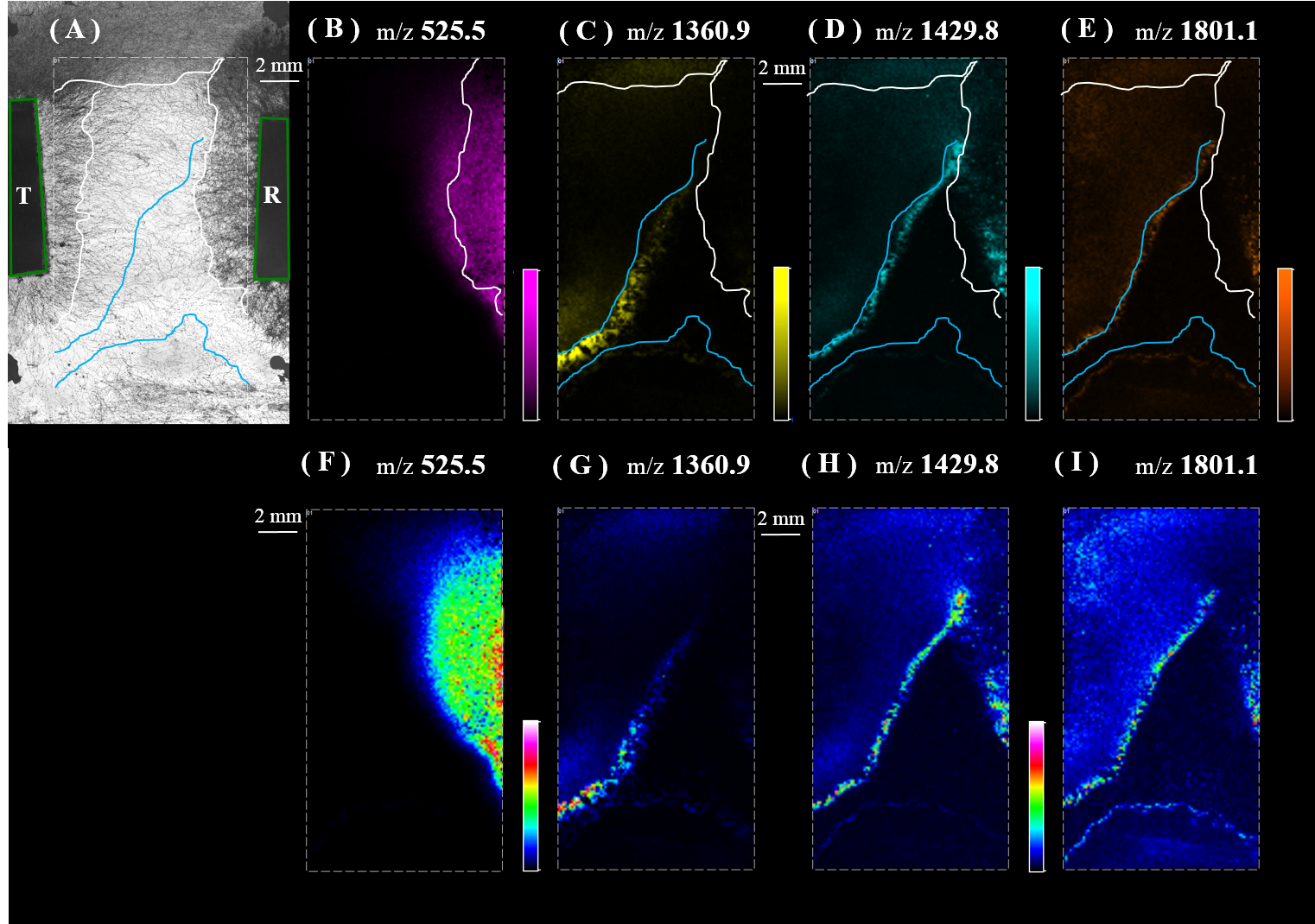


S4: Single ion images of *R.solani* (R) specific metabolites derived during physical interaction with *T.atroviride* (T). (A) Light microscopic image showing points of inoculation (green tetragons), the outer rim of dense hyphal growth for both species (white lines) and borders for features detected by MSI (blue lines). Spatial distributions of metabolites with (B) m/z 525.5, (C) m/z 1360.9, (D) m/z 1429.8 and (E) m/z 1801.1 are visualized. Images (F), (G), (H) and (I) highlight the respective ion intensity distributions.

**Supplemental Figure 5 and 6**

As suppression of ionization represents a significant issue in MALDI MSI, intensity distributions of ions in the hyphae free zone (non-interacting assay, S5) and in a particular area free of detected metabolites (interacting assay, S6) are shown.


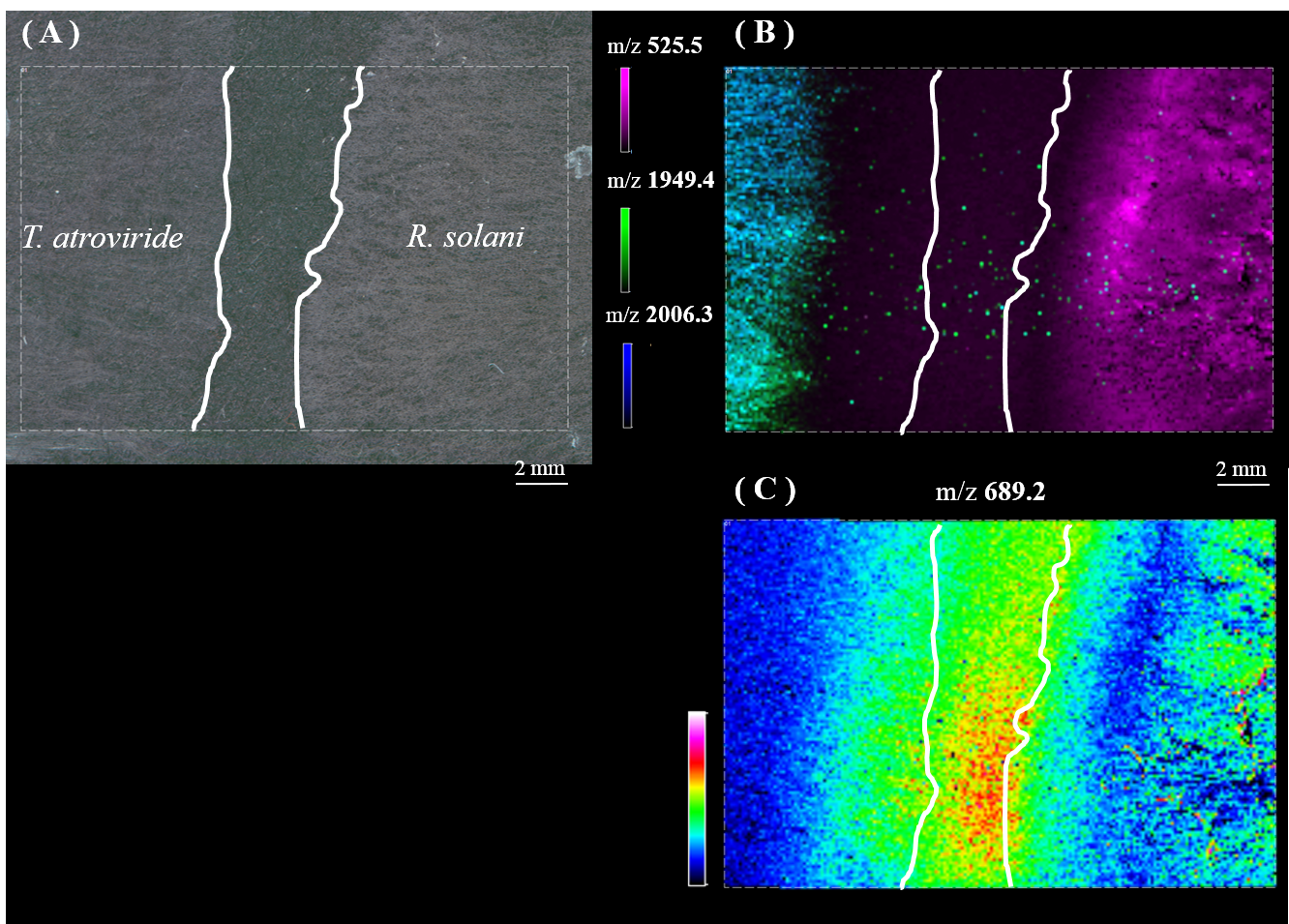


S5: MALDI MSI of physically non-interacting *T.atroviride* and *R.solani* hyphae. (A) Light microscopic image showing non-interacting hyphae of *T.atroviride* and *R. solani*. White lines mark the outer rim of hyphal growth. (B) Distribution of selected m/z values representing characteristics for *T. atroviride* (m/z 1949.4 and 2006.3) and *R. solani* (m/z 525.5). (C) Intensity distribution of a background ion (m/z 689.2) from the growth medium in the hyphae free zone indicating no potential ion suppression in the particular area.


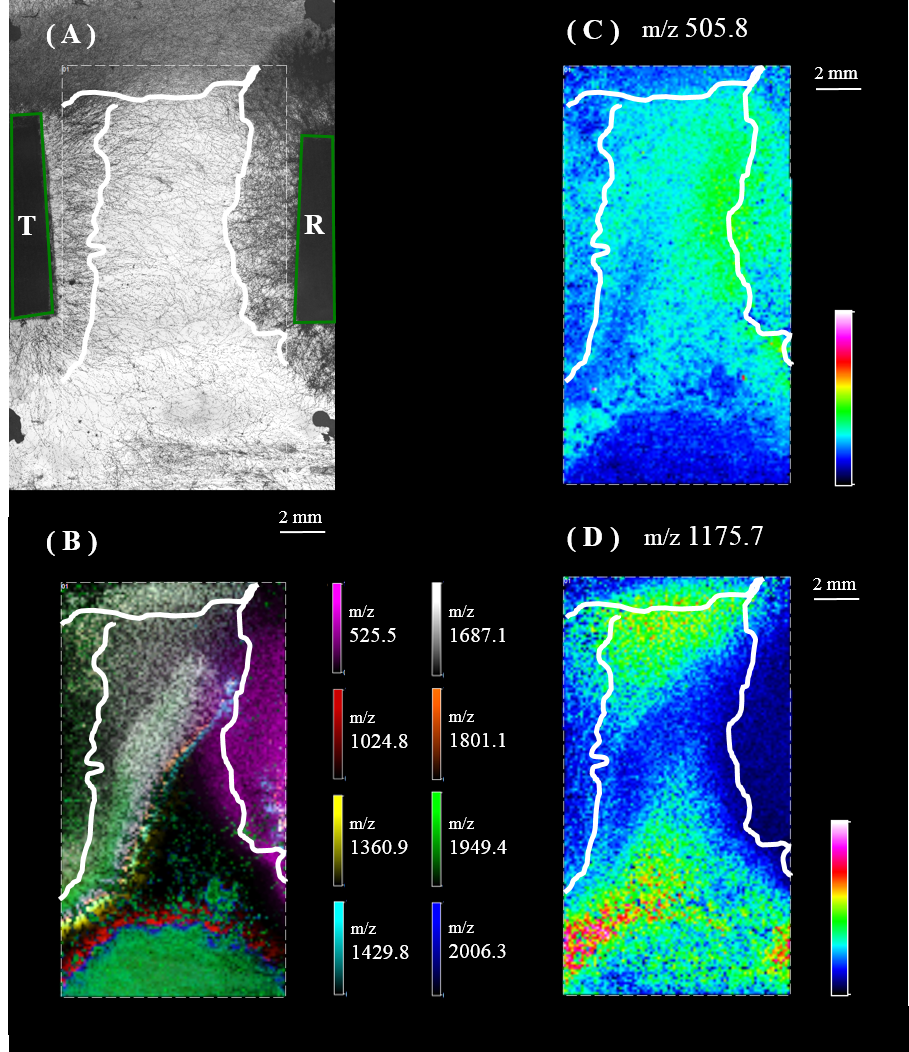


S6: Disproval of ion suppression during MALDI MSI of physically interacting *T.atroviride* and *R.solani* hyphae. (A) Light microscopic image of interacting *T.atroviride* (T) and *R.solani* (R) hyphae. White lines mark the outer rim of hyphal growth for both species. Green tetragons show the points of inoculation. (B) Molecular distributions of selected secondary metabolites characteristic for *T.atroviride* and *R.solani* localized by MALDI MSI. Intensity distributions of (C) a minor abundant metabolite with m/z 505.8. and (D) a background ion derived from PDA with m/z 1175.7 disproving potential suppression of ionization in the particular area.

**Supplemental Figure 7**

Profile mass spectra of separately grown *R.solani* and *T.atroviride* were measured to exclude the detected secondary metabolites from being produced by non-interacting fungi but rather being stress-released during the mycoparasite-host interaction.


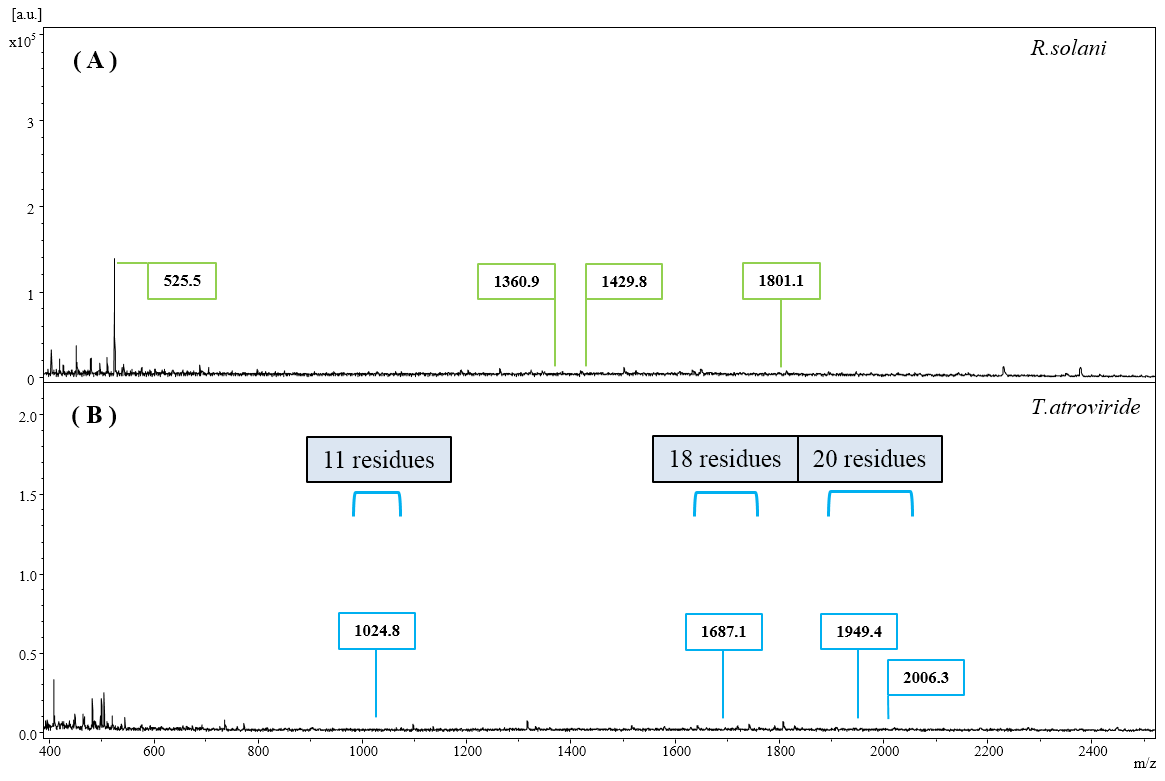


S7: Individual mass profiles of separately grown (A) *R.solani* and (B) *T.atroviride* (ROI 400-2600 Da). These mass spectra exhibit absence of characteristic signals of metabolites being released by (A) *R.solani* (1360.9/1429.8/1801.1; in green) and (B) *T.atroviride* (1024.8/1687.1/1949.4/2006.3; in blue) during mycoparasite-host interaction.
